# Association between albumin-to-globulin ratio and the risk of overall survival in advanced non-small cell lung cancer patients with anlotinib treatment: a retrospective cohort study

**DOI:** 10.1186/s12890-023-02574-6

**Published:** 2023-07-25

**Authors:** Jinzhan Chen, Congyi Xie, Yimin Yang, Shuwen Yang, Jinxian Huang, Feiyang Ye, Zhenyang Lin, Lin Tong, Jiaxin Liu

**Affiliations:** 1 Department of Pulmonary Medicine, Zhongshan Hospital (Xiamen), Fudan University, Xiamen, 361000 Fujian China; 2 Xiamen Clinical Research Center for Cancer Therapy, Xiamen, 361000 Fujian China; 3 Department of Vascular Surgery, Zhongshan Hospital, Fudan University, Shanghai, 200032 China; 4 College of Computer and Data Science, Fuzhou University, Fuzhou, 350108 Fujian China; 5 Department of Thoracic Surgery, Zhongshan Hospital (Xiamen), Fudan University, Xiamen, 361000 Fujian China; 6 Department of Pulmonary and Critical Care Medicine, Zhongshan Hospital, Fudan University, Shanghai, 200032 China

**Keywords:** Lung cancer, Anlotinib, Albumin, Globulin, Overall survival

## Abstract

**Objective:**

Researches about the association between serum albumin-to-globulin ratio (AGR) and the prognosis of lung cancer are limited. We aimed to investigate the relationship between AGR and overall survival (OS) in patients with advanced non-small-cell lung cancer (NSCLC) treated with anlotinib.

**Methods:**

A retrospective cohort study was conducted on 196 advanced NSCLC patients with anlotinib treatment between June 1, 2018 and June 1, 2021. The exposure was AGR, calculated by baseline serum albumin / (serum total protein - serum albumin). The outcome was OS, defined as the period from the date of initial treatment with anlotinib to death or the last follow-up. The univariate and multivariate linear regression models and generalized additive models (GAM) were used to analyze the relationship between AGR and OS. The Kaplan-Meier method was used to analyze the OS.

**Results:**

After adjusting for potential confounders, a non-linear relationship was observed between AGR and OS, which had an inflection point of 1.24. The hazard ratio and the confidence intervals on the left and the right sides of the inflection point were 13.05 (0.52 to 327.64) and 0.20 (0.07 to 0.57), respectively. It suggested that AGR was positively associated with OS when AGR was larger than 1.24, for every 1 unit increase in AGR, the risk of death lowered approximately by 80%.

**Conclusions:**

The relationship between AGR and the OS for advanced NSCLC patients with anlotinib is non-linear. AGR level is an independent protective factor for OS in advanced NSCLC patients who received anlotinib therapy.

**Supplementary Information:**

The online version contains supplementary material available at 10.1186/s12890-023-02574-6.

## Introduction

Recently, the epidemiological survey released that lung cancer is still the most diagnosed type of cancer, accounting for 20% of global cancer-related mortality [1, 2]. Non-small-cell lung cancer (NSCLC) is the major type of lung cancer, accounting for approximately 85% of all primary lung cancers [3]. At present, in addition to the traditional treatment methods, the rapid development of targeted therapy and immunotherapy has significantly improved the survival of lung cancer patients. The application of antiangiogenic drugs in lung cancer patients’ treatment is considered to have good therapeutic effects. Anlotinib is a selective receptor tyrosine kinase inhibitor that targets vascular endothelial growth factor receptors, fibroblast growth factor receptors, platelet-derived growth factor receptor-α and c-Kit [4]. Based on the overall survival benefit observed in a randomized, double-blind, multicenter, phase III trial (ALTER0303) [5], the China National Medical Products Administration approved anlotinib for the treatment of third-line advanced NSCLC. However, not all patients will benefit from anlotinib, thus, it is particularly of utmost importance to find prognostic biomarkers, which are simple, inexpensive and affordable, to select suitable patients. Albumin and globulin have attracted wide attention as non-invasive prognostic factors of tumors. Albumin can be used to reflect the nutritional and systemic inflammatory status of cancer patients and can be used as a prognostic marker for diverse cancers [6–10]. Globulin, as one of the main cortisol-binding proteins, can participate in immune and inflammatory responses [11]. As a combination of albumin and globulin, the albumin-to-globulin ratio (AGR) reflects both nutritional status and inflammatory response [12], which has been reported to be associated with the prognosis of several cancers [10, 13–16].We suggest that AGR is a credible predictive parameter for several reasons. Firstly, a lower AGR level can be attributed to reduced albumin levels and/or elevated globulin levels, encompassing both significant unfavorable predictors and potentially enhancing the accuracy of predictions compared to individual parameters. Secondly, the advantage of AGR lies in its relative insensitivity to conditions such as dehydration and fluid retention, which can cause fluctuations in albumin and globulin levels. However, so far, there is no study that have reported the relationship between AGR and overall survival (OS) of advanced NSCLC patients treated with anlotinib. Therefore, we retrospectively studied the relationship between AGR and OS with the hypothesis that AGR level can be an independent protective factor for OS in advanced NSCLC patients treated with anlotinib.

## Methods

### Study design and population

To explore the relationship between AGR and OS in patients diagnosed with advanced NSCLC and treated with anlotinib, we conducted a retrospective cohort study. All patients included in this study were admitted to the Department of Pulmonary and Critical Care Medicine of Zhongshan Hospital, Fudan University between June 1, 2018 and June 1, 2021. Based on the inclusion criteria as follows: (a) Patients with either histologically or cytologically confirmed diagnosis of NSCLC; (b) Patients with anlotinib treatment; (c) There was no concurrent malignancy or history of a second primary malignancy, a total of 306 patients were successive enrolled initially. In addition, patients who met the exclusion criteria as follows: (a) Patients without available follow-up data and records; (b) Patients treated with anlotinib for less than one cycle (12 mg, 10 mg or 8 mg once daily, 2 weeks on/ 1 week off); (c) Patients were not at clinical stage III and IV (d) Patients with the Eastern Cooperative Oncology Group Performance Status (ECOG PS) score higher than one, were not included in the final analysis.

Data regarding demographics, comorbidities, clinicopathological characteristics, gene mutations, distant metastases, previous treatment information and complete follow-up information were extracted from medical records or obtained from telephone follow-up. Laboratory values, including serum albumin and total protein, were collected at baseline within a week prior to the first dose of anlotinib.

The AGR value was defined as serum albumin / (serum total protein - serum albumin), as previously described [16]. OS was defined as the period from the date of initial treatment with anlotinib to death or the last follow-up. Patient without smoking history was defined as never smoker. Clinical stages were determined by IASLC (International Association for the Study of Lung Cancer) 8th edition lung cancer staging system. Informed consent was waived due to the retrospective nature of the study. The cutoff data for patients’ follow-up was November 1, 2021.

### Statistical analysis

The first step of data analysis was to present the baseline data distribution of patients enrolled in this study in different AGR groups (Tertile). We expressed continuous variables as means ± standard deviations (normal distribution) or medians (quartile) (skewed distribution). We expressed categorical variables as frequencies or percentages. We used chi-square test (categorical variables), One-Way ANOVA (normal distribution) and Kruskal-Wallis H (skewed distribution) to calculate the differences among different AGR groups.

The second step of data analysis could be summarized as follows: (a) Is there a relationship between AGR and OS, is it linear or non-linear? (b) What factors interfered with or modified the relationship between them? (c) What was the independent effect on AGR and OS when we exclude the effects of these potential confounders or modifiers? In accordance with the above analytical principles, univariate and multivariate linear regression models were used to assess the associations between AGR and OS. As suggested by the recommendations of STROBE statement [17], we built three models including an unadjusted model, a model adjusted to demographics and a fully-adjusted model. For the fully-adjusted model, the adjusted variables were correlated covariates that may influence OS and/or AGR as reported in previous studies [6, 14, 16]. In addition, the subgroup analyses were executed using stratified linear regression models after fully adjusted. Tests for effect modification by subgroups used the interaction terms between subgroup indicators, followed by the likelihood ratio test. OS among AGR tertiles was assessed using the Kaplan-Meier method and log-rank tests.

To ensure the robustness of the data analysis results, we conducted the following sensitivity analyses: (a) We converted the AGR into a categorical variable by tertile. The aim was to verify the results of AGR as a continuous variable and to observe the possibility of nonlinearity. (b) Linear regression is a linear model that is calculated based on the fact that the relationships between the independent variables and the dependent variables were linear. However, the relationships between the dependent variables and the independent variables are often nonlinear in biomedical data analysis. Thus, we used a generalized additive model (GAM) to explore the nonlinear relationships. (c) If the relationship between AGR and OS is nonlinear, a two-piecewise linear regression model would be constructed to figure out the threshold effect of the AGR on OS according to the smoothing plot. A recursion algorithm was used to determine the saturation level of AGR, at which the relationship between AGR and OS began to change and became significant. The inflection point moved along a predefined interval and detected the inflection point that gave the maximum model likelihood. We determined the best fit model based on the p value of log-likelihood ratio tests. When the p value of log-likelihood ratio tests was greater than 0.05, there is no difference between the linear regression model and the two-piecewise linear model, and the linear model can be used to fit the relationship between AGR and OS. On the contrary, when the p value was less than 0.05, it is considered that the above-mentioned models were significantly different, and we used the two-piecewise linear model to clarify the relationship between AGR and OS.

All the analyses were performed with the statistical software package R (http://www.R-project.org, The R Foundation) and EmpowerStats (http://www.empowerstats.com, X&Y solutions, Inc., Boston, MA). P values less than 0.05 (two-side) were considered statistically significant.

## Results

### The enrollment of participants

**Fig. 1:**
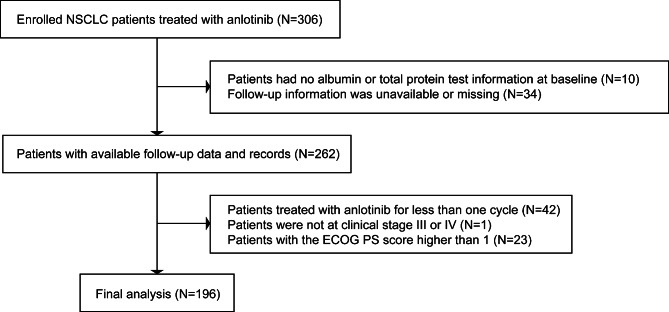
Flowchart of the study participants enrollment

In total, 306 NSCLC patients treated with anlotinib were admitted to the Department of Pulmonary and Critical Care Medicine of Zhongshan Hospital, Fudan University between June 1, 2018 and June 1, 2021. Of these, 110 patients were excluded, including 10 patients who missed baseline data, 34 patients who lost to follow-up, 42 patients who were treated with anlotinib for less than one cycle, 23 patients with a high ECOG PS score (> 1) and 1 patient who was not at clinical stage III and IV. Finally, 196 selected participants were enrolled for data analysis (Fig. 1).

### Baseline clinical characteristics

The median follow-up time was 7.64 (range 0.59 to 32.98) months, and 97 patients died (49.49%). Baseline characteristics of selected participants according to tertile of AGR are shown in Table 1. In general, the average age was 60.44 ± 10.14 years old, and about 64.29% were male. No statistically significant differences were detected in neutrophil, gender, never smoker, hypertension, *ALK* rearrangement, *EGFR* mutation, tumor stage, ECOG PS score, number of metastases, number of previous treatment lines, previous targeted therapy, previous radiotherapy, previous immunotherapy and anlotinib monotherapy (all p values > 0.05). Compared with low level AGR group, patients had a significantly lower age, leukocyte, platelets and globulin in the high level AGR group. The opposite pattern was observed in albumin. In the population with low level AGR, adenocarcinoma accounted for the majority, and less subjects received more than two chemotherapy lines previously compared with those in the high level AGR group.

**Table 1:** Baseline characteristics of participants according to the tertiles of AGR (n = 196). Values are mean ± SD or n (%)

Characteristics	AGR (Total)	AGR tertile			p-value
		Low (0.70–1.34)	Middle (1.35–1.65)	High (1.65–2.45)	
No. of participants	196	65	64	67	
Age, years	60.44 ± 10.14	62.78 ± 9.55	61.86 ± 9.31	56.82 ± 10.56	0.001
Male	126 (64.29%)	45 (69.23%)	37 (57.81%)	44 (65.67%)	0.384
Never smoker	127 (64.80%)	38 (58.46%)	45 (70.31%)	44 (65.67%)	0.364
Hypertension	66 (33.67%)	22 (33.85%)	25 (39.06%)	19 (28.36%)	0.432
Leukocyte, 10^9^/L	7.07 ± 2.57	7.70 ± 2.79	7.05 ± 2.17	6.48 ± 2.60	0.023
Neutrophil, 10^9^/L	4.97 ± 2.21	5.35 ± 2.40	4.99 ± 1.91	4.58 ± 2.24	0.132
Lymphocyte, 10^9^/L	1.31 ± 0.55	1.42 ± 0.56	1.33 ± 0.55	1.19 ± 0.54	0.055
Platelets, 10^9^/L	256.67 ± 98.46	292.65 ± 109.65	258.56 ± 98.37	219.97 ± 71.43	< 0.001
Albumin, g/L	41.84 ± 4.65	38.15 ± 4.66	43.37 ± 3.56	43.98 ± 3.23	< 0.001
Globulin, g/L	28.51 ± 4.97	33.18 ± 3.84	28.97 ± 2.56	23.52 ± 2.33	< 0.001
*ALK* rearrangement					0.395
No	136 (69.39%)	41 (63.08%)	45 (70.31%)	50 (74.63%)	
Yes	3 (1.53%)	1 (1.54%)	2 (3.12%)	0 (0.00%)	
Unknown	57 (29.08%)	23 (35.38%)	17 (26.56%)	17 (25.37%)	
*EGFR* mutation					0.200
No	83 (42.35%)	27 (41.54%)	31 (48.44%)	25 (37.31%)	
Yes	57 (29.08%)	15 (23.08%)	16 (25.00%)	26 (38.81%)	
Unknown	56 (28.57%)	23 (35.38%)	17 (26.56%)	16 (23.88%)	
Histology					0.033
Adenocarcinoma	124 (63.27%)	49 (75.38%)	42 (65.62%)	33 (49.25%)	
Squamous cell carcinoma	47 (23.98%)	9 (13.85%)	15 (23.44%)	23 (34.33%)	
Others	25 (12.76%)	7 (10.77%)	7 (10.94%)	11 (16.42%)	
Tumor stage					0.853
III	30 (15.31%)	11 (16.92%)	10 (15.62%)	9 (13.43%)	
IV	166 (84.69%)	54 (83.08%)	54 (84.38%)	58 (86.57%)	
ECOG PS score					0.348
0	136 (69.39%)	41 (63.08%)	45 (70.31%)	50 (74.63%)	
1	60 (30.61%)	24 (36.92%)	19 (29.69%)	17 (25.37%)	
Number of metastases					0.955
< 3	153 (78.06%)	50 (76.92%)	50 (78.12%)	53 (79.10%)	
≥ 3	43 (21.94%)	15 (23.08%)	14 (21.88%)	14 (20.90%)	
Number of previous treatment lines					0.070
< 3	107 (54.59%)	40 (61.54%)	38 (59.38%)	29 (43.28%)	
≥ 3	89 (45.41%)	25 (38.46%)	26 (40.62%)	38 (56.72%)	
Number of previous chemotherapy lines					0.029
≤ 2	153 (78.06%)	54 (83.08%)	54 (84.38%)	45 (67.16%)	
> 2	43 (21.94%)	11 (16.92%)	10 (15.62%)	22 (32.84%)	
Previous targeted therapy	69 (35.20%)	21 (32.31%)	19 (29.69%)	29 (43.28%)	0.222
Previous radiotherapy	35 (17.86%)	12 (18.46%)	9 (14.06%)	14 (20.90%)	0.587
Previous immunotherapy	32 (16.33%)	11 (16.92%)	10 (15.62%)	11 (16.42%)	0.980
Anlotinib monotherapy	156 (79.59%)	53 (81.54%)	52 (81.25%)	51 (76.12%)	0.685

*Continuous variable was obtained by Kruskal-Wallis rank sum test. If the count variable had a theoretical number < 10, the probability was calculated accurately using Fisher’s exact test

### The univariate analysis of OS

The results of univariate analysis were shown in Table 2. The results of univariate analysis showed that female, albumin and AGR (Hazard ratio (HR) = 0.47, 95% CI: 0.26 to 0.84, p = 0.0105) were positively associated with OS. We also found that age, never smoker, hypertension, *ALK* rearrangement, *EGFR* mutation, histology, tumor stage, ECOG PS score, number of metastases, number of previous treatment lines, number of previous chemotherapy lines, previous targeted therapy, previous radiotherapy, previous immunotherapy, anlotinib monotherapy, leukocyte, neutrophil, lymphocyte, platelets and globulin were not associated with OS.

**Table 2:** The results of univariate analysis of overall survival (n = 196). Values are mean ± SD or n (%)

Covariates	Statistics	Hazard ratio (95% CIs), p-value
Age, years	60.44 ± 10.14	1.01 (0.99, 1.04) 0.1624
Female	70 (35.71%)	0.60 (0.38, 0.92) 0.0201
Never smoker	69 (35.20%)	1.08 (0.72, 1.64) 0.7038
Hypertension	66 (33.67%)	1.11 (0.74, 1.68) 0.6070
Leukocyte, 10^9^/L	7.07 ± 2.57	1.04 (0.96, 1.11) 0.3226
Neutrophil, 10^9^/L	4.97 ± 2.21	1.05 (0.96, 1.14) 0.2963
Lymphocyte, 10^9^/L	1.31 ± 0.55	0.90 (0.63, 1.28) 0.5478
Platelets, 10^9^/L	256.67 ± 98.46	1.00 (1.00, 1.00) 0.2259
Albumin, g/L	41.84 ± 4.65	0.92 (0.88, 0.95) < 0.0001
Globulin, g/L	28.51 ± 4.97	1.01 (0.97, 1.05) 0.7345
AGR	1.52 ± 0.33	0.47 (0.26, 0.84) 0.0105
*ALK* rearrangement		
No	136 (69.39%)	1.0
Yes	3 (1.53%)	0.89 (0.21, 3.71) 0.8743
Unknown	57 (29.08%)	1.00 (0.64, 1.55) 0.9866
*EGFR* mutation		
No	83 (42.35%)	1.0
Yes	57 (29.08%)	0.90 (0.56, 1.46) 0.6706
Unknown	56 (28.57%)	0.92 (0.57, 1.50) 0.7518
Histology		
Adenocarcinoma	124 (63.27%)	1.0
Squamous cell carcinoma	47 (23.98%)	0.89 (0.55, 1.45) 0.6430
Others	25 (12.76%)	0.81 (0.43, 1.53) 0.5158
Tumor stage		
III	30 (15.31%)	1.0
IV	166 (84.69%)	1.67 (0.91, 3.07) 0.0964
ECOG PS score		
0	136 (69.39%)	1.0
1	60 (30.61%)	1.17 (0.75, 1.82) 0.4810
Number of metastases		
<3	153 (78.06%)	1.0
≥3	43 (21.94%)	1.49 (0.94, 2.37) 0.0886
Number of previous treatment lines		
<3	107 (54.59%)	1.0
≥3	89 (45.41%)	1.08 (0.73, 1.61) 0.6975
Number of previous chemotherapy lines		
≤2	153 (78.06%)	1.0
>2	43 (21.94%)	0.93 (0.58, 1.49) 0.7676
Previous targeted therapy	69 (35.20%)	1.15 (0.76, 1.74) 0.5034
Previous radiotherapy	35 (17.86%)	0.72 (0.42, 1.24) 0.2364
Previous immunotherapy	32 (16.33%)	1.08 (0.63, 1.84) 0.7910
Anlotinib monotherapy	156 (79.59%)	0.93 (0.55, 1.59) 0.7998

**Fig. 2:**
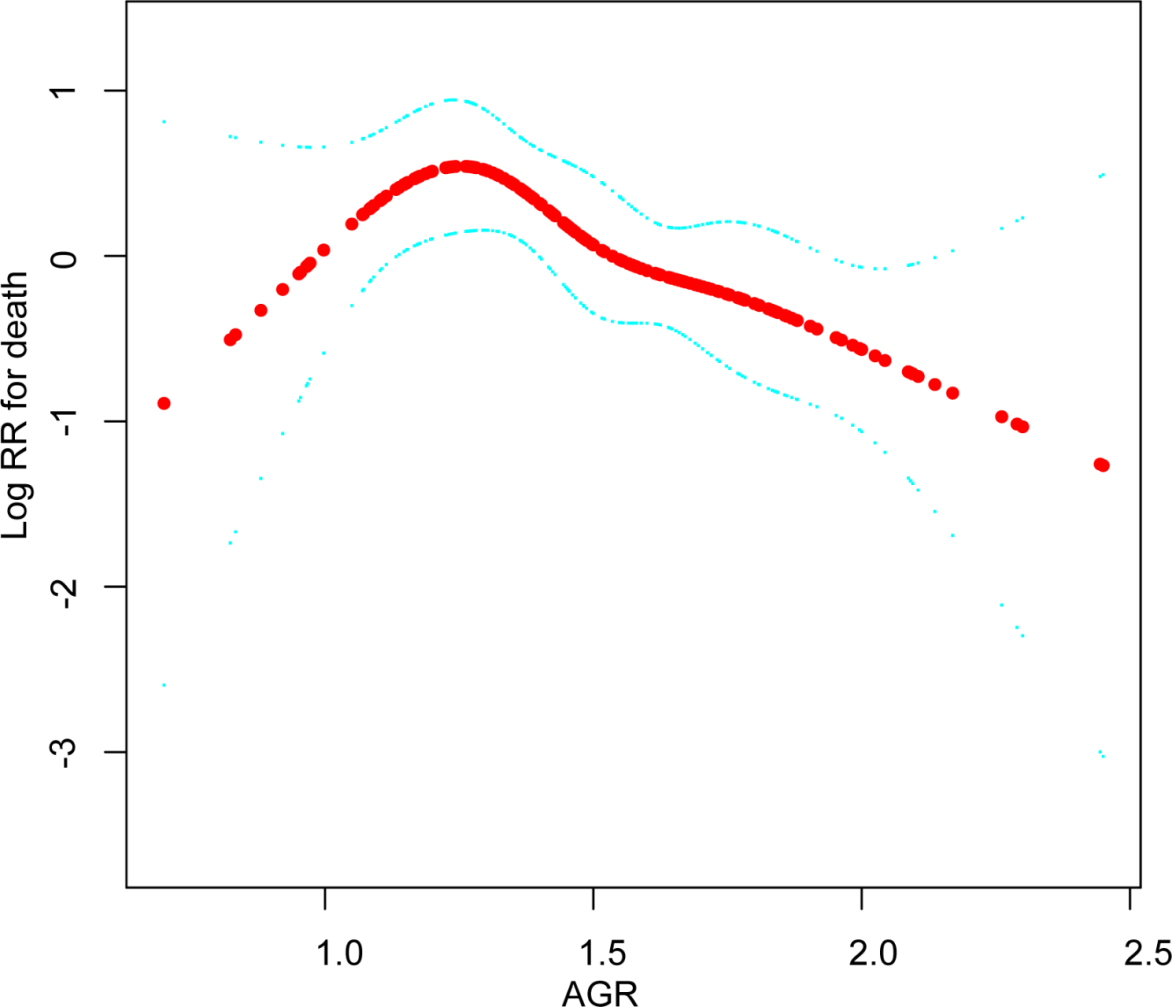
Illustrated curved line relation between AGR and overall survival

### The relationship between AGR and OS

The results of multivariate linear regression model were shown in Table 3. The non-adjusted model showed that for each additional unit increase of AGR, there was approximately a 53% lower risk of death (95% CI: 0.26 to 0.84, p = 0.0105). In minimally adjusted model (only adjusted for age, gender and never smoker), the results did not have notable changes (HR = 0.53, 95% CI: 0.29 to 0.96, p = 0.0365). After adjusting other potential covariates, AGR in the fully-adjusted model was still positively associated with OS (HR = 0.40, 95% CI: 0.18 to 0.88, p = 0.0230). For sensitivity analysis, we also converted the AGR into categorical variable by tertile and figured out p for trend. In fully-adjusted model, compared with the reference of low group, the estimated reduce of the risk of death in the middle and high group were 35% and 51%, respectively. The p for the trend was 0.0277.

**Table 3:** Multiple Cox regression analysis of AGR in patients with advanced NSCLC treated with anlotinib

Exposure	Crude model	Minimally adjusted model	Fully adjusted model
	Hazard ratio (95% CIs), p-value	Hazard ratio (95% CIs), p-value	Hazard ratio (95% CIs), p-value
AGR	0.47 (0.26, 0.84) 0.0105	0.53 (0.29, 0.96) 0.0365	0.40 (0.18, 0.88) 0.0230
AGR (tertiles)			
Low	Ref	Ref	Ref
Middle	0.58 (0.36, 0.95) 0.0293	0.58 (0.35, 0.96) 0.0332	0.65 (0.36, 1.16) 0.1410
High	0.52 (0.32, 0.84) 0.0080	0.55 (0.33, 0.91) 0.0187	0.49 (0.26, 0.92) 0.0273
p for trend	0.0073	0.0164	0.0277

Crude model adjusted for: None

Minimally adjusted model adjusted for: Age; Gender; Never smoker

Fully adjusted model adjusted for: Age, years; Gender; Never smoker; Hypertension; ALK rearrangement; EGFR mutation; Histology; Tumor stage; ECOG PS score; Number of metastases; Number of previous treatment lines; Number of previous chemotherapy lines; Previous targeted therapy; Previous radiotherapy; Previous immunotherapy; Anlotinib monotherapy; Leukocyte; Neutrophil; Lymphocyte; Platelets. Restricted cubic spline was applied

### The analyses of non-linear relationship

In this present study, we analyzed the non-linear relationship between AGR and OS (Fig. 2). The result of smooth curve through the generalized additive model showed that the relationship between AGR and OS was non-linear (after fully adjusted). We compared linear regression model (fitting the relationship between AGR and OS by a linear regression) and two-piecewise linear regression model (fitting the relationship between AGR and OS by a curve) (Table 4). The p for log-likelihood ratio test was less than 0.05, which indicated that the two-piecewise linear regression model should be used to fit the relationship between AGR and OS. By two-piecewise linear regression model and recursive algorithm, we figured out the inflection point was 1.24. On the right of inflection point (AGR > 1.24), the hazard ratio, 95% CI and p value were 0.20, 0.07 to 0.57 and 0.0025, respectively. However, on the left side of the inflection point (AGR ≤ 1.24), we did not observe an association between AGR and OS (HR = 13.05, 95% CI: 0.52 to 327.64, p = 0.1183).

**Table 4:** The results of the two-piecewise linear regression model

AGR	No. of participants	Hazard ratio (95% CIs)	p-value
Fitting model by standard linear regression	196	0.60 (0.26, 1.36)	0.2191
Fitting model by two-piecewise linear regression			
≤ 1.24	41	13.05 (0.52, 327.64)	0.1183
> 1.24	155	0.20 (0.07, 0.57)	0.0025
p for log-likelihood ratio test			0.0240

Adjusted for: Age; Gender; Never smoker; Hypertension; ALK rearrangement; EGFR mutation; Histology; Tumor stage; ECOG PS score; Number of metastases; Number of previous treatment lines; Number of previous chemotherapy lines; Previous targeted therapy; Previous radiotherapy; Previous immunotherapy; Anlotinib monotherapy; Leukocyte; Neutrophil; Lymphocyte; Platelets. Restricted cubic spline was applied

### The results of Kaplan-Meier analyses

**Fig. 3:**
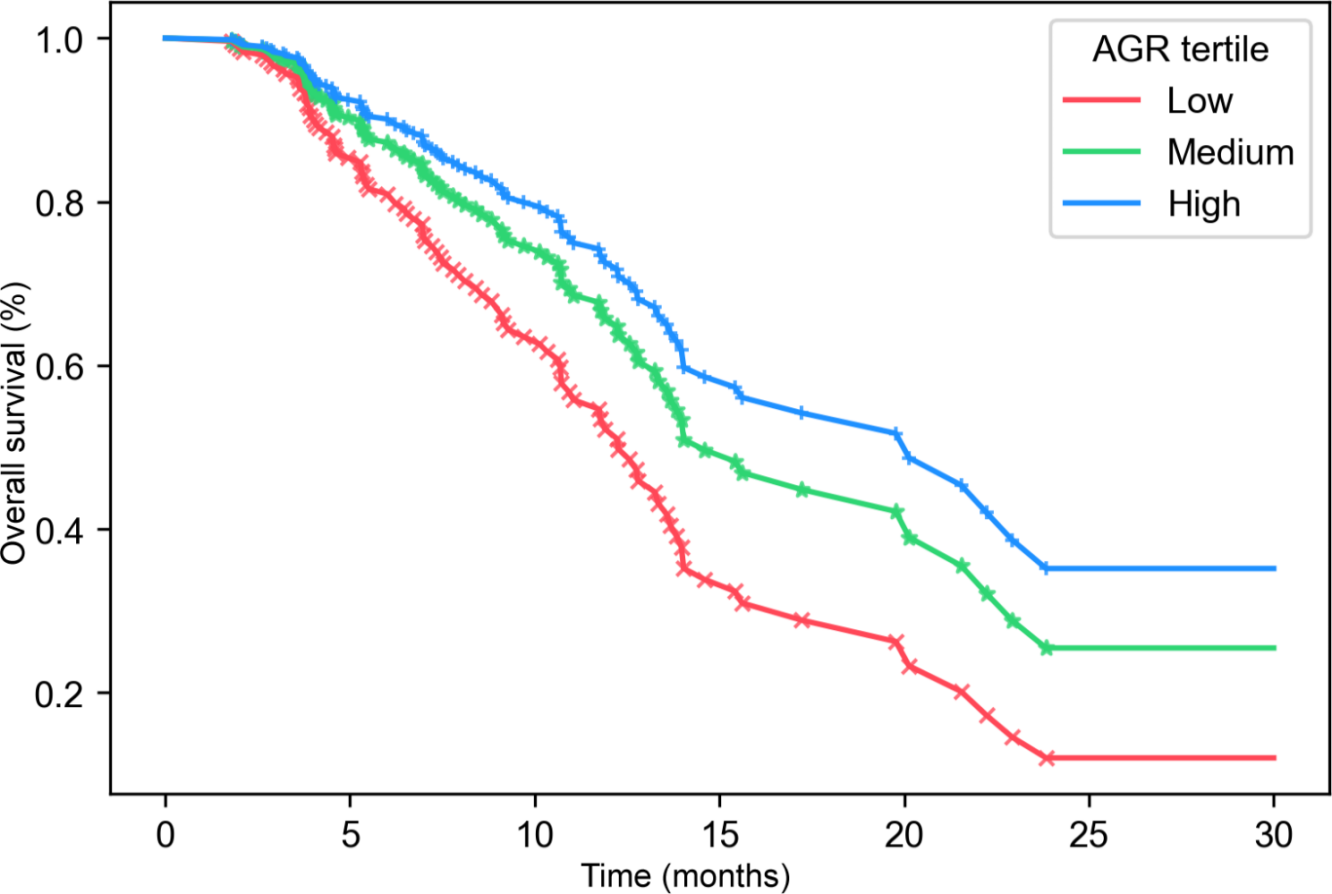
Kaplan-Meier survival curves for overall survival were compared among different AGR groups

Figure 3 showed the Kaplan-Meier curves of overall survival in advanced NSCLC patients treated with anlotinib, stratified by AGR groups. The median OS in low, middle and high AGR groups were 12.26, 14.59 and 20.13 months, respectively (after fully adjusted). These differences between different groups were statistically significant (the p value of the log-rank test was less than 0.05).

### The results of subgroup analyses

**Fig. 4:**
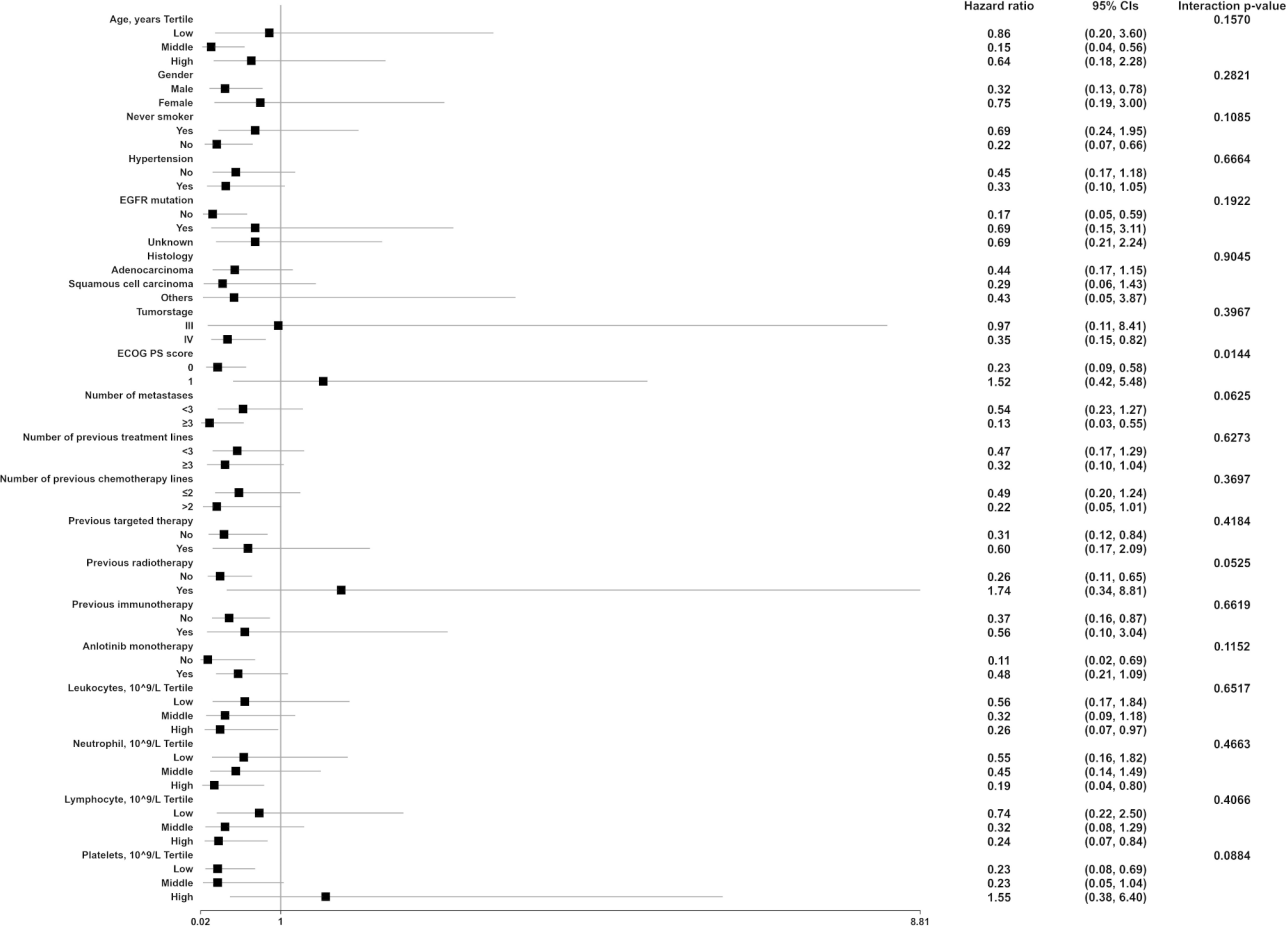
Subgroup analysis of the association between AGR and overall survival

As is shown in Fig. 4, the tests of interactions were significant for ECOG PS score (p for interaction = 0.0144), while the tests of interaction were not statistically significant for other covariants (all the p values for interactions were larger than 0.05) (*ALK* rearrangement was not included in subgroup analysis because the sample size in the subgroup was less than 10). For patients with the ECOG PS score = 0, a high AGR was associated with favorable prognosis, demonstrating a 77% lower risk of death (HR = 0.23, 95% CI: 0.09 to 0.58) as AGR increased by 1 unit. For patients with the ECOG PS score = 1, the association between AGR and risk of death had a trend but was not statistically significant (HR = 1.52, 95% CI: 0.42 to 5.48).

## Discussion

Although several papers have suggested a relationship between AGR and prognosis in patients with various types of cancers [6, 10, 13, 14, 16, 18], this evidence in NSCLC remains limited. As we know, our study is the first to explore AGR as a prognostic marker in advanced NSCLC patients with anlotinib treatment. In the present study, generalized linear model (GLM) and GAM model were used to elucidate the relationship between AGR and OS. As shown in the fully adjusted model, AGR was positively associated with OS in advanced NSCLC patients treated with anlotinib. In addition, we converted AGR into a categorical variable and the same positive correlation trend was observed. The non-isometric changes of hazard ratio indicated that there may be a non-linear relationship between AGR and OS. Therefore, we further used GAM and two-piecewise linear regression model to explore the relationship between AGR and OS, and clarified it was non-linear, and the correlation between AGR and OS was different on the left and right sides of the inflection point (AGR = 1.24). This result suggested that AGR was not correlated with OS on the left side of the inflection point (AGR ≤ 1.24), but AGR was positively correlated with OS when AGR was higher than 1.24, and for every 1 unit increase in AGR, the risk of death was reduced by 80%, which means AGR may be a promising prognostic indicator in clinical practices.

Current studies focusing on the relationship between inflammation and malignant tumor have found that the host systemic inflammatory response and tumor microenvironment are both crucial in tumor initiation, progression and metastasis [19, 20]. This has also been confirmed in lung cancer [21–23]. Albumin and globulin are major serum proteins, which can reflect the systemic inflammatory response. Albumin regulates systemic inflammatory reaction and exert an influence on antioxidant effects. Low albumin levels have been confirmed to be a useful prognostic tool for lung cancer [24, 25]. Globulin increases with the accumulation of acute-phase proteins and immunoglobulins, which reflect immune and inflammatory states [26]. As mentioned above, both albumin and globulin could be involved in cancer progression in various ways and play crucial roles. Based on this, the AGR, which accounts for the values of both albumin and globulin, has been used as one of the inflammatory parameters to assess the systemic inflammatory status of the host [27]. Meanwhile, several studies have attempted to explore the prognostic value of AGR in malignant tumors. Our study results were consistent with those reported by Suh et al. who conducted a large retrospective study in generally healthy adults and found that low AGR was a risk factor for cancer morbidity and mortality [16]. Hua Zhang et al. suggested that the preoperative AGR might be a predictor of postoperative chemotherapy efficacy in patients with stage II and III NSCLC [6]. Ping Lu et al. also found that the OS of metastatic NSCLC patients with high AGR was longer than that with low AGR, and suggested that AGR can serve as a prognostic tool for metastatic NSCLC [10]. Although most of studies mentioned above have suggested that there was linear association for AGR and OS, but they did not address their nonlinearity, and did not perform the subgroup analysis. In our study, we used GAM and two-piecewise linear regression model, which could deal with non-parametric smoothing and would fit a regression spline to the data, to further discover the non-linear relationship between AGR and OS. Therefore, the contribution of this study was the discovery of a threshold effect on the linear relationship between AGR and OS. The inflection point we calculated by the recursive algorithm was 1.24. The results showed that not all levels of AGR correlated with OS. Only when AGR level was higher than 1.24, the risk of death was significantly reduced by 80% for every 1 unit increase in AGR. This means that only when inflammation improves to a certain level or immune status improves to a certain level does the risk of death correspondingly reduced. When AGR falls below 1.24, it may not exhibit a significant independent association with OS in advanced NSCLC patients receiving anlotinib treatment. One possible explanation for this result is that when AGR is lower than 1.24, it may reflect a state of compromised liver function, which is often associated with advanced stages of lung cancer. In such cases, there are likely multiple indicators and factors contributing to the prognosis of patients, and the influence of AGR alone may be diminished. Further studies to find suitable prognostic factors in those specific patients are needed.

Subgroup analysis is quite important for a scientific study. This analysis will help us to better understand the independent association of AGR and OS from known risk factors. In the present study, we used age, gender, never smoker, hypertension, *EGFR* mutation, histology, tumor stage, ECOG PS score, number of metastases, number of previous treatment lines, number of previous chemotherapy lines, previous targeted therapy, previous radiotherapy, previous immunotherapy, anlotinib monotherapy, leukocyte, neutrophil, lymphocyte and platelets as stratification variables, of which only ECOG PS score was identified. Hence, our study demonstrated that the linearly decreasing trend between AGR and risk of death occurred only in participants with ECOG PS score = 0 group. One possible explanation is that patients with ECOG PS score = 1 in our study had a higher number of metastases (Supplement Table 1), indicating that they may have higher tumor burden, which is generally associated with worse outcomes in cancer patients. And there may be multiple factors contributing to the prognosis of this group of patients, which may need further study with larger sample size to elucidate.

To our knowledge, we are the first to show that AGR may be a clinical indicator associated with OS for advanced NSCLC patients treated with anlotinib. Since AGR is a simple, inexpensive and easily available biomarker, and is part of routinely administered laboratory tests, it is economically beneficial for its widespread clinical application. Despite the above-mentioned strengths, there are some limitations in our study. First, this study is a retrospective cohort study, and thus, it provides only weak evidence of associations between AGR and OS; it is hard to distinguish cause and effect. Second, the manifestations of possible selection bias, detection bias, and analysis bias might be confounded due to the nature of retrospective study. Third, as the absence of prior research in investigating the association between AGR and OS specifically in patients with ECOG PS score = 1 group, further investigations to understand the underlying mechanisms and determine if this relationship is consistent across different patient populations are needed. Forth, the small sample size may have affected the statistical power and generalizability of our findings. It is important to interpret the results with caution and recognize that they may not be representative of the larger population of patients with advanced lung cancer. Furthermore, the patient characteristics and treatment modalities in our study may not fully represent the heterogeneity seen in the overall population of patients with advanced lung cancer. Therefore, caution should be exercised when extrapolating our results to other patient cohorts or making clinical decisions solely based on our findings.

## Conclusions

The relationship between AGR and OS for advanced NSCLC patients treated with anlotinib is non-linear. AGR is positively correlated with OS when AGR is higher than 1.24. These findings further expand the potential role of AGR as a prognostic predictor in advanced NSCLC patients treated with anlotinib.

## Electronic supplementary material

Below is the link to the electronic supplementary material.

Supplementary Material 1

## Data Availability

The datasets used and/or analyzed during the current study available from the corresponding author on reasonable request.
