# Supplementary material for: Association between albumin-to-globulin ratio and the risk of overall survival in advanced non-small cell lung cancer patients with anlotinib treatment: a retrospective cohort study
Source: BMC Pulm Med. 2023 Jul 25;23:275. doi: 10.1186/s12890-023-02574-6 (PMC10369733; doi:10.1186/s12890-023-02574-6)
Supplement: Supplementary file 1 — Supplementary Material 1 [file 12890_2023_2574_MOESM1_ESM.doc]

**Supplement Table 1** Baseline characteristics of participants according to ECOG PS score. Values are mean ± SD or n (%).

| ECOG PS score | 0 | 1 | p-value |
| --- | --- | --- | --- |
| No. of participants | 136 | 60 |  |
| Age, years | 59.46 ± 10.11 | 62.67 ± 9.92 | 0.041 |
| Male | 86 (63.24%) | 40 (66.67%) | 0.644 |
| Never smoker | 86 (63.24%) | 41 (68.33%) | 0.491 |
| Hypertension | 48 (35.29%) | 18 (30.00%) | 0.470 |
| Leukocyte, 10^9/L | 7.04 ± 2.46 | 7.15 ± 2.83 | 0.780 |
| Neutrophil, 10^9/L | 4.94 ± 2.09 | 5.03 ± 2.48 | 0.797 |
| Lymphocyte, 10^9/L | 1.31 ± 0.56 | 1.32 ± 0.54 | 0.848 |
| Platelets, 10^9/L | 249.90 ± 93.07 | 272.03 ± 108.97 | 0.147 |
| Albumin, g/L | 42.27 ± 4.07 | 40.88 ± 5.67 | 0.053 |
| Globulin, g/L | 27.96 ± 4.54 | 29.73 ± 5.69 | 0.021 |
| AGR | 1.56 ± 0.32 | 1.43 ± 0.35 | 0.014 |
| *ALK* rearrangement |  |  | 0.011 |
| No | 100 (73.53%) | 36 (60.00%) |  |
| Yes | 0 (0.00%) | 3 (5.00%) |  |
| Unknown | 36 (26.47%) | 21 (35.00%) |  |
| *EGFR* mutation |  |  | 0.305 |
| No | 62 (45.59%) | 21 (35.00%) |  |
| Yes | 39 (28.68%) | 18 (30.00%) |  |
| Unknown | 35 (25.74%) | 21 (35.00%) |  |
| Histology |  |  | 0.196 |
| Adenocarcinoma | 86 (63.24%) | 38 (63.33%) |  |
| Squamous cell carcinoma | 36 (26.47%) | 11 (18.33%) |  |
| Others | 14 (10.29%) | 11 (18.33%) |  |
| Tumor stage |  |  | 0.347 |
| III | 23 (16.91%) | 7 (11.67%) |  |
| IV | 113 (83.09%) | 53 (88.33%) |  |
| Number of metastases |  |  | 0.010 |
| <3 | 113 (83.09%) | 40 (66.67%) |  |
| ≥3 | 23 (16.91%) | 20 (33.33%) |  |
| Number of previous treatment lines |  |  | 0.485 |
| <3 | 72 (52.94%) | 35 (58.33%) |  |
| ≥3 | 64 (47.06%) | 25 (41.67%) |  |
| Number of previous chemotherapy lines |  |  | 0.236 |
| ≤2 | 103 (75.74%) | 50 (83.33%) |  |
| >2 | 33 (24.26%) | 10 (16.67%) |  |
| Previous targeted therapy | 48 (35.29%) | 21 (35.00%) | 0.968 |
| Previous radiotherapy | 22 (16.18%) | 13 (21.67%) | 0.355 |
| Previous immunotherapy | 24 (17.65%) | 8 (13.33%) | 0.451 |
| Anlotinib monotherapy | 110 (80.88%) | 46 (76.67%) | 0.500 |

*Continuous variable was obtained by Kruskal-Wallis rank sum test. If the count variable had a theoretical number <10, the probability was calculated accurately using Fisher’s exact test.
